# Review: Correlation between bladder obstruction with bladder function and erectile dysfunction in mice

**DOI:** 10.1016/j.amsu.2022.103294

**Published:** 2022-01-25

**Authors:** Charles Martamba Hutasoit, Andi Wardihan Sinrang, Mochammad Hatta, Haerani Rasyid, Hendry Lie

**Affiliations:** a Dept of Urology, Siloam Hospitals Kebon Jeruk, Jl. Perjuangan No.Kav.8, RT14/RW10, Kebon Jeruk, DKI Jakarta, 11530, Indonesia; b Dept of Physiology, Faculty of Medicine, Universitas Hasanuddin, Jl. Sahabat Raya No. 3, Talamanrea Indah, Kota Makassar, Sulawesi Selatan, Indonesia; c Dept of Molecular Biology and Immunology for Infectious Disease, Faculty of Medicine, Universitas Hasanuddin, Jl. Sahabat Raya No. 3, Talamanrea Indah, Kota Makassar, Sulawesi Selatan, Indonesia; d Dept of Internal Medicine, Faculty of Medicine, Universitas Hasanuddin, Jl. Sahabat Raya No. 3, Talamanrea Indah, Kota Makassar, Sulawesi Selatan, Indonesia; e Dept of Surgery, Faculty of Medicine, Universitas Pelita Harapan, Jl. Siloam No. 6, Bencongan, Kelapa Dua, Tangerang, Banten, 15811, Indonesia

**Keywords:** Bladder obstruction, Bladder dysfunction, Erectile dysfunction

## Abstract

Bladder obstruction, including due to benign prostate enlargement (BPH), will trigger its anatomy and physiological function changes. Men with BPH have a 6 times higher risk of erectile dysfunction than those without BPH. Morphological and functional changes in subjects with partial bladder outlet obstruction (pBOO) occur differently depending on the duration of pBOO that has been experienced. The underlying pathophysiology of BPH is closely related to erectile dysfunction. Anatomically, functionally, and psychologically changes due to BPH will also have an impact on sexual function. Chronic pBOO causes lower urinary tract symptoms (LUTS) through a complex pathophysiological pathway. LUTS and bladder obstruction can lead to erectile dysfunction. The severity of LUTS and sexual dysfunction is inversely related to the quality of life. The treatment of LUTS symptoms will also enhance sexual function.

## Highlights

• Bladder morphology and functional changes are depending on the partial bladder outlet obstruction (pBOO) duration that is experienced and also have an impact on sexual function.
• Ischemia leads to increased production of reactive oxygen species (ROS), malondialdehyde (MDA). Free radicals from ischemia-reperfusion injury are one of the major causes of bladder obstruction.
• Obstruction of the bladder neck nerve may be associated with impaired sensory axons.
• PBOO play a modulating role alongside key mechanisms such as vascular changes, altered CCSM tone/contractility, phosphodiesterase-dependent pathway.
• Chronic pBOO are increased risk of infection through complex pathophysiological pathway involving nitric oxide guanosine monophosphate and RhoA/Rho-kinase, metabolic syndrome, autonomic hyperactivity, pelvic ischemia, psychological factors, imbalance of sex hormones, and inflammatory pathways.

## 1 Introduction

Nerve, muscle, and psychological disorders, infections, and obstructions can cause urinary excretion disorders. Bladder obstruction may be caused by prostate enlargement and this pathological condition will trigger the anatomy and physiological function changes. Increased urination frequency and nocturia due to hyperactivity of bladder emptying are the symptoms of bladder dysfunction. Other symptoms are urgency, dysuria, incontinence, or urinary retention that reduces the quality of life [1].

Ejaculation occurs through two phases: (1) emission, a phase of semen production that contains sperm, and (2) expulsion, the phase of semen excretion from the urethral meatus. Ejaculation problems that are often faced are ejaculating too fast or ejaculating too late [2]. The condition in which a man is unable to maintain an erection sufficient for satisfying sexual purposes is named erectile dysfunction. Sexual function disorders occur with a complex pathophysiology and are caused by multifactorial etiology, such as nervous and vascularization system disorders, side effects of drugs or surgical procedure [3], and aging process [4]. More than half of the elderly male population experience it with varying degrees of severity. Comorbid diseases, such as heart disease, diabetes, hypertension, and psychological disorders also increase the risk of erectile dysfunction [5].

Much researches and development of appropriate therapy have been carried out on animals as the research subjects. The bladder function studies were carried out on humans and mice as subjects to understand the physiological control of urinary incontinence and its dysfunction pathophysiology. Several studies have used mice, rats, rabbits, and guinea pigs to generate BOO conditions, whose structural and physiological changes in the bladder wall are similar to those observed in men with BPH.

## 2 Discussion

The prevalence of benign prostate hyperplasia (BPH) is quite high, but the pathophysiological mechanism of urinary disorders caused by bladder outlet obstruction is still unknown how it causes erectile dysfunction [6,7]. Half of the male population in the 5th decade of age experience BPH, and a quarter suffer from LUTS [8].

The results of a urological study conducted by Elbadawi et al. to determine bladder morphology in the geriatric group showed that bladder morphology and functional changes are depending on the partial bladder outlet obstruction (pBOO) duration that is experienced by the subject. pBOO is characterized by shortened bladder contractions interval and hypertrophied detrusor muscle to compensate for complete bladder emptying despite the obstruction [9]. This has been observed in animals.

Studies show that morphological and functional changes in subjects with pBOO occur differently depending on the duration of pBOO that has been experienced, in the majority of cases subjects suffer from pBOO for 4–6 weeks. The first two weeks are the acute phase. In the chronic phase, the bladder will go through two phases: compensated and decompensated. In the compensatory phase, the detrusor muscle increases strength to overcome the increased resistance. But even though bladder mass increases, the bladder contractility function is still normal so it can still be resolved. In the decompensated phase, bladder mass increases but bladder wall contractility decreases and emptying function getting worsens. The end of this phase is characterized by a low bladder capacity to accommodate urine and a fibrotic bladder wall [6,7,10].

The typical histologic appearance of the pBOO bladder wall is characterized by hypertrophy, thickening of the bladder wall, and a significant increase in bladder weight. Cystometrogram observations of mice with pBOO also revealed bladder hypertrophy [11]. These characteristics are similar to those found in patients with BPH. Electron microscopy studies have shown that muscle hypertrophy and hyperplasia increase collagen deposition and loss of parasympathetic nerve terminals, thereby producing an unstable electrical state and resulting in an irregular distribution of current patterns. These findings support experimental observations that hypertrophic bladder contracts with lower threshold stimulation and weaker contractions compared with normal bladders. The number of mast cells present in the mucosa or submucosa is known to increase in mice with pBOO, this makes the bladder muscle weaker [12,13].

During bedtime, bladder capacity decreases, and the amount of urine excreted increases in both humans and mice. The results of a study by Kitta et al. on mice that had undergone proximal urethral ligation surgery to induce pBOO conditions showed that the frequency of mice urinating began to decrease significantly from 3 months to 1 year after pBOO surgical intervention. There was a significant decrease in the frequency of urination during the day 6 months after pBOO surgery intervention. The bodyweight of the mice increased after a year of pBOO surgery, this was due to the increase in soft and adipose tissue [11]. Through an unknown mechanism, obesity is a risk factor of nocturia [14].

Mice with BOO showed a nearly 2-fold increase in bladder weight to bodyweight ratio. Bladder mass was increased a week after obstruction intervention, this was worsened 3–5 weeks after the surgical procedure. Similarly, mice and rabbits experienced increased blood flow after 24 h of obstruction surgery, which could be the first stimulus for hypertrophy[[15], [16], [17]]. These events occur due to stretching of the bladder wall muscles, causing thickening of the epithelium, muscle, and serous lining and increased synthesis and deposition of collagen. Once bladder function is compensated, blood flow tends to decrease. In the chronic phase, there will be more areas of hypoxia-reperfusion in the smooth muscle layer. During the voiding process, there is a straining process that causes an ischemia-reperfusion cycle. In addition, ischemia leads to increased production of reactive oxygen species (ROS), malondialdehyde (MDA). Free radicals from ischemia-reperfusion injury are one of the major causes of bladder obstruction. A 4-week study of female BOO mice had edema and lymphocytic infiltration in the lamina propria, and hypoxia in the urothelium, lamina propria, and detrusor [18].

The pBOO technique, procedure, and model are distinct from the acute BOO model in humans with BPH. The most common method of inducing pBOO is by reducing the diameter of the urethra using sutures around the urethra via a transperitoneal approach. Typically, with or without a urethral catheter, the internal diameter is 0.7 mm, and the external diameter is 1.1 mm. Some researchers report that the mortality rate is typically 15%. Levin et al. reported that in the pBOO model, some bladders have close to normal detrusor hypertrophy function, whereas others were decompensated, exhibiting high intravesical pressure and large residual urine volume. They classified the pBOO model not based on the obstruction severity, but in based on the bladder weight [19,20].

The pBOO modeling technique with a transperitoneal approach by performing a midline incision and the tissue around the bladder to open the abdomen. This technique is prone to injury that affects bladder function. Pelvic organs are dissected, and trauma can cause nerve disturbances and bladder deformity, which can affect bladder function. The healing process of the abdominal incision also occurs with inflammation and adhesion of the bladder to the abdominal wall, both factors induce bladder dysfunction and are usually not part of BPH condition. To avoid this drawback of the transperitoneal pBOO model, Zhang et al. reported a urethral with perineal ligation model. This procedure does not require a lower abdominal incision and does not irritate the bladder, periurethral nerves, and blood vessels. This model shows similar results to the transperitoneal model concerning histologic changes and cystetric data [21,22].

Experiments in mice with pBOO also support our suggestion that mild obstruction of the bladder neck nerve may be associated with impaired sensory axons that supply both organs. Therefore, we provide additional evidence that pBOO, the main cause of LUTS in men, also induces nerve fibers innervating damages of the bladder and contributing to the neurogenic development that caused erectile dysfunction. We emphasize that alterations in neural pathways triggered by PBOO are not suggested to be the main cause of LUTS-ED comorbidity, but rather play a modulating role alongside key mechanisms such as vascular changes, altered CCSM tone/contractility, phosphodiesterase-dependent pathways [23,24].

The underlying pathophysiology of BPH is closely related to erectile dysfunction. Anatomically, functionally, and psychologically changes of the bladder will also have an impact on sexual function [25]. Men with BPH have a 6 times higher risk to experience erectile dysfunction than those without BPH [26]. Complications of chronic pBOO are increased risk of infection causing lower urinary tract symptoms (LUTS) through a complex pathophysiological pathway involving nitric oxide guanosine monophosphate and RhoA/Rho-kinase, metabolic syndrome, autonomic hyperactivity, pelvic ischemia, psychological factors, imbalance of sex hormones, and inflammatory pathways [27]. One of the most frequently complained symptoms of LUTS is nocturia [28]. Many studies have shown that LUTS can be complicated by erectile dysfunction.

The results of research by Matsuda on 108 men of varying ages showed that the majority of subjects with erectile dysfunction had experienced symptoms of nocturia since before. The majority of the group experienced LUTS along with erectile dysfunction, the rest experienced LUTS first then followed by erectile dysfunction [5]. The severity of LUTS can be assessed using a scoring system, for example, American Urological Association Symptom Index [29]. Sexual dysfunction potentially occurs in someone with severe LUTS (score 20 or more), obesity, and old age. Researches have shown that there is a decrease in blood flow to the bladder and prostate as men age [30]. The severity of LUTS and sexual dysfunction is inversely related to the quality of life (QoL) of the sufferer. And adequate LUTS treatments will also slowly enhance sexual function.

Research by Matsuda et al. on mice showed erectile dysfunction occurred 16 weeks after mice had bladder obstruction surgery. It is concluded that LUTS and bladder obstruction can trigger erectile dysfunction [5]. Nocturia or at least 2 times urination at night is one of the LUTS symptoms which is suggested to be an early predictor factor before it develops into erectile dysfunction. Studies using transrectal color Doppler ultrasonography have shown that bladder perfusion decreases with the increase of urination frequency at night [32].

BPH which can complicate LUTS needs to be treated as soon as possible before it complicates and causes erectile dysfunction. The first line of pharmacological treatment for BPH is an alpha-blocker, such as tamsulosin. However, it is known that this drug can cause retrograde ejaculation and anejaculation. Surgical treatment also causes erectile dysfunction as its side effect [25]. Transurethral resection of the prostate (TURP) is the standard surgical procedure for BPH treatment. However, up to 32% of patients experience side effects such as erectile dysfunction, and up to 72% experience ejaculatory disorders after undergoing the surgical procedure [33]. The pathophysiological mechanism that may be behind the occurrence of retrograde ejaculation with or without decreased ejaculation is the accidental resection of the paracollicular and supracollicular tissue in the verumontanum and the decrease in prostate tissue volume after resection. Furthermore, the use of the high-frequency energy generated near the prostatic capsule can cause neuropraxia injury to the nearby neuromuscular bundles, resulting in erectile dysfunction [34,35].

## 3 Conclusion

LUTS and bladder obstructions that are caused by any condition can lead to erectile dysfunction. The underlying pathophysiology of BPH is closely related to erectile dysfunction. Anatomically, functionally, and psychologically changes that occur due to BPH will have an impact on sexual function. The severity of LUTS and sexual dysfunction is inversely related to the quality of life. And adequate treatment of LUTS will also enhance sexual function.

## Funding

There is no sponsor for this study, all funding from my self.

## Ethical approval

---

## Author contribution

Charles Martamba Hutasoit author. Prof. Andi Wardihan Sinrang co-author, Prof. Mochammad Hatta co-author. Prof Haerani Rasyid co-author. Hendry Lie co-author.

## Consent

---

## Guarantor

Prof. Mochammad Hatta.

## Declaration of competing interest

There is no conflicts of interest for this study.
